# Supplementary material for: A multicenter study comparing the bacterial reduction on flexible endoscopes without a working channel between UV-C light disinfection versus standard endoscope Washer Disinfection: a randomized controlled trial
Source: Antimicrob Resist Infect Control. 2024 Oct 26;13:128. doi: 10.1186/s13756-024-01486-2 (PMC11515285; doi:10.1186/s13756-024-01486-2)
Supplement: Supplementary file 1 — Supplementary material 1. [file 13756_2024_1486_MOESM1_ESM.docx]

**A multicenter study comparing the bacterial reduction on flexible endoscopes without a working channel between UV-C light disinfection versus standard Endoscope Washer Disinfection: a randomized controlled trial**

Y. Halmans MD^1^, D.J. Wellenstein MD PhD^1^, M. Romijn BSc^1^, A.J.M. van Bemmel MD LLM^2^, H. van den Berge MD PhD^3^, R.A. Scheeren MD PhD^4^, J.S. Kalpoe MD PhD^5^, R. Klont MD^6^, J.H. van Zeijl MD PhD^7^, H. Sikkema MD^8^, S.M. Euser MD PhD^9^, J. Hopman MD PhD^10^, R.P. Takes MD PhD Prof^1^, G.B. van den Broek MD PhD^1^

1. Department of Otorhinolaryngology and Head and Neck Surgery, Radboud University Medical Center Nijmegen, P/O Box 9101, Nijmegen, The Netherlands

2. Department of Otorhinolaryngology and Head and Neck Surgery, Medical Spectrum Twente, P/O 50000, Enschede, The Netherlands

3. Department of Otorhinolaryngology and Head and Neck Surgery, Medical Center Leeuwarden, P/O 888, Leeuwarden, The Netherlands

4. Department of Otorhinolaryngology, Spaarne Hospital, P/O 417, Haarlem, The Netherlands

5. Department of Medical Microbiology, Spaarne Hospital, P/O 417, Haarlem, The Netherlands

6. Department of Medical Microbiology, Medical Spectrum Twente, P/O 50000, Enschede, The Netherlands

7. Department of Medical Microbiology, Medical Center Leeuwarden, P/O 888, Leeuwarden, The Netherlands

8. Department of Hopsital Pharmacy. Medical Center Leeuwarden, P/O 888 8901 BR, Leeuwarden, The Netherlands

9. Department of Epidemiology and Infection Prevention, Regional Public Health Laboratory Kennemerland, Haarlem, The Netherlands

10. Department of Medical Microbiology, Radboud University Medical Center Nijmegen, P/O Box 9101, Nijmegen, The Netherlands

Corresponding author: Yana Halmans, Radboud University Medical Center Nijmegen, P/O Box 9101, Nijmegen, The Netherlands. Tel: +3124 361 1111, e-mail: [yana.halmans@radboudumc.nl](mailto:yana.halmans@radboudumc.nl)

Manuscript word count: 2498

**Abstract**

**Background:** To prevent cross-contamination between patients, adequate reprocessing is necessary when using flexible endoscopes(FEs) without a working channel. The current reprocessing process using an Endoscope Washer Disinfector(EWD) is time-consuming. Ultraviolet light group C(UV-C) exposition is an alternative and fast disinfection method and has previously been shown to adequately reduce Colony Forming Units(CFUs) on FEs without a working channel. The objective of this study was to examine whether UV-C light is as effective in reducing CFUs on contaminated FEs without a working channel compared to the EWD.

**Methods:** FEs without a working channel were collected in three different Otorhinolaryngology Departments in the Netherlands. After pharyngolaryngoscopy, a manual pre-cleaning with tap water was performed and a culture was collected by rolling the distal 8-10 cm of the FE over an agar plate. Next, the FE was randomly assigned to be disinfected with UV-C light(D60) or the EWD(gold standard). After disinfection, another culture was taken. The primary outcome was microbiological contamination, defined by Colony Forming Units(CFU).

**Results:** 600 FEs without a working channel were randomized. After clinical use and manual pre-cleaning, 239/300(79.7%) FEs in the UV-C group and 262/300(87.3%) FEs in the EWD group were contaminated(i.e., >0 CFU). FEs without culture confirmed contamination were excluded from further analysis. After UV-C light disinfection, 195/239(81.6%) FEs showed 0 CFUs, compared to 187/262(71.4%) FEs disinfected with the EWD(p<0.01). A multivariate logistics regression analysis showed an increased odds of 0 CFUs when using UV-C light(OR 1.83, 95% CI 1.19-2.79; p<0.01), conditional on participating hospitals and types of FE.

**Conclusions:** UV-C light disinfection of FEs without a working channel appears more effective in reducing CFUs compared to the EWD and might be a good alternative disinfection method.

**Trial registration:** Not applicable

**Key words**

Ultraviolet light C, UV-C, disinfection, Endoscope Washer Disinfector, Colony Forming Units, Flexible endoscope without a working channel

**Background**

Disinfecting medical devices is essential for preventing healthcare-related infections(1). Within otorhinolaryngology(ORL), flexible endoscopes(FEs) without a working channel are indispensable in the diagnostic pathway. FEs can become contaminated with blood and microorganisms, so adequate reprocessing is essential to reduce pathogen transmission(1, 2).

FEs without a working channel come into contact with mucous membranes without entering sterile tissue or the vasculature and are considered semi-critical devices requiring high-level disinfection(3, 4). High-level disinfection eradicates mycobacteria, lipid or medium-sized viruses, fungal spores, nonlipid or small viruses, vegetative bacteria, and some bacterial endospores during relatively short disinfection exposure times(3, 5, 6).

Multiple guidelines have been developed for reprocessing FEs. These include the American Society for Gastrointestinal Endoscopy(ASGE), the European Society for Gastrointestinal Endoscopy and European Society of Gastroenterology Nurses and Associates(ESGE-ESGENA) and the Healthcare Infection Control Practices Advisory Committee(HICPAC)(7-9). National guidelines exist, such as the Dutch Flexible Endoscopes Cleaning and Disinfection Steering Committee(SFERD) and manufacturers’ guidelines(10). Although most of these guidelines agree on the methods used for reprocessing, differences exist, resulting in a lack of international consensus. The methods described, mostly including an Endoscope Washer Disinfector(EWD), are time-consuming and use chemicals and water. Other disinfection methods, such as ultraviolet light group C(UV-C), could be an alternative(11).

Disinfection with UV-C light was successfully used in 1936 to disinfect air in operating rooms to prevent postoperative infections(12). UV-C light can inactivate bacteria, such as Methicillin-resistant Staphylococcus aureus(MRSA), Clostridium difficile spores, and vancomycin-resistant Enterococcus(VRE)(13-18). Previous studies investigating the effect of UV-C light in reprocessing FEs without a working channel are promising, showing a 10^7^ reduction of colony forming units(CFUs)(11). Recently, we conducted a single-center study to compare the CFU reduction of FEs without a working channel with UV-C light disinfection to the EWD(19). There was no difference in CFU reduction between UV-C light disinfection and the EWD. A multicenter study was initiated to validate these results. The objective of this study was to investigate CFU reduction on contaminated FEs without a working channel using UV-C light compared to the EWD in a multicenter setting.

**Methods**

*Trial design and study objects*

This is a randomized controlled trial of parallel groups(1:1) conducted at three ORL departments in the Netherlands: Medical Spectrum Twente(Enschede), Medical Center Leeuwarden(Leeuwarden), and Spaarne Hospital(Hoofddorp). The research ethics committee of all participating centers decided that this study would be carried out per the applicable legislation concerning reviewal by an accredited research ethics committee, such as Medical Research involving Human Subjects Act and Medical Treatment Contracts Act(file number 2021-9837).

The eligibility criteria were FEs without a working channel after being used in a clinical setting. From March until August 2022, 600 FEs without a working channel were collected after pharyngolaryngoscopy. No selection was made between infectious and non-infectious patients. FEs used included the Video Naso-Pharyngo-Laryngoscope[VNL9-CP](PENTAX Nederland B.V., Dodewaard, The Netherlands), Fiber Naso-Pharyngo-Laryngoscopes[FNL10-RP3](PENTAX Nederland B.V., Dodewaard, The Netherlands) and pediatric Fiber Naso-Pharyngo-Laryngoscopes[FNL7-RP3](PENTAX Nederland B.V., Dodewaard, The Netherlands). FEs without a working channel showing no contamination(i.e., 0 CFUs) after clinical use and manual pre-cleaning were excluded.

*Randomization*

FEs were randomly assigned to the UV-C light (group 1) or EWD group(group 2) by two researchers(YH, MR). To standardize the study process as much as possible, culture collections were evaluated by two researchers(YH, MR) or a laboratory analyst from the microbiology laboratories of the participating hospitals.

*Interventions*

The manual pre-cleaning in this study differs from the one recommended by the UV-C light disinfector manufacturer and standard hospital protocol to evaluate the necessity of chemicals in the disinfection process. Manual pre-cleaning usually includes water and chemicals to remove visible debris. However, the manual pre-cleaning in this study consisted of moistening a gauze pad with tap water and moving it rotationally from proximal to distal over the FE to remove visible debris.

After pre-cleaning, a culture(culture 1) was taken. Then, the FE was disinfected with UV-C light or with the EWD. Afterwards, another culture(culture 2) was taken. For patient safety, all FEs were finally pre-cleaned and disinfected with the EWD, as protocolled by the hospital.

*Microbiological culture collection*

The microbiological cultures were collected by rolling the distal 8-10cm of the shaft and tip of the FE over a Plate Count Agar+additives(Balis Laboratorium BV, Boven-Leeuwen, The Netherlands) until the entire circumference had touched the plate. The additives included tween, lecithin, sodium thiosulfate pentahydrate, and agar. Sterile tweezers were used to fix the FE to prevent partial lifting(Appendix 1). The cultures were incubated at 36°C for 72 hours. A detailed protocol is given in Appendix 2.

*Endoscope Washer Disinfector*

The EWD disinfects several medical devices simultaneously using water and chemicals. The duration of the reprocessing depends on the brand and type, with, in this study, a minimum of 22 minutes. The EWD and chemicals used varied between hospitals. Medical Spectrum Twente used the standard program from the WD440PT(Wassenburg Medical Nederland, Dodewaard, The Netherlands). Medical Center Leeuwarden used the standard program from the ED-Flow 4(Getinge AB, Göteborg, Sweden). Spaarne Hospital used the standard program from the STEELCO® EW2S endoscope washer disinfectors(PMT Partners Medische Techniek, Alblasserdam, The Netherlands). All EWDs used accompanying chemicals.

Guidelines provide different recommendations for drying processes. Most guidelines only recommend drying a FE with a working channel. The ASGE states that the exterior of the endoscope should be completely dried using a clean, lint-free cloth(8). The ESGE-ESGENA guideline recommends drying all external parts with compressed air(9). The drying process after the EWD in this study consisted of wiping off the access water using a microfiber cloth(Appendix 2).

*The D60 UV-C Disinfector*

The D60(UV Smart Technologies B.V., Rijswijk, The Netherlands) disinfects the outer surfaces of channelless medical devices in 60 seconds, operating at a wavelength of 100-280nm(peak at 253.7nm). According to the manufacturer’s internal research, a reduction in microorganisms of at least a log-4 is achieved(unpublished data, research available upon request at the manufacturer). According to previous studies, exposure to UV-C light in the applied UV-C dose does not damage the surface of the FE (11).

The endoscope is placed in a glass holder made of quartz, allowing the UV-C light to reach the endoscope without shadowing. The disinfection chamber is completely sealed off, preventing the UV-C light from reaching the user for the user’s safety. The UV-C light is automatically switched off when the disinfection cycle is complete. The disinfection process does not require chemicals or liquids other than those used for pre-cleaning. Pre-cleaning is necessary since UV-C light cannot penetrate dirt, debris, and grime(20).

*Outcomes*

The primary outcome was microbiological contamination, which was evaluated by performing a CFU count. Cultures showing no contamination after clinical use and manual pre-cleaning(culture 1) were excluded from further statistical analysis since since assessing the disinfection effectiveness in these FEs is impossible.

*Sample size*

The sample size was not calculated since the prevalence of contamination on FEs without a working channel after clinical usage is unknown. We therefore chose to base the sample size on expert opinion.

*Statistical methods*

Statistical significance was determined with a Chi-square test. An univariate and multivariate logistic regression model was performed using the disinfection method as a predictor with additional corrections for endoscope type and participating hospital. Data were analyzed using SPSS software, version 25(IBM Corporation, Armonk, NY, USA). A p-value of <0.05 was considered statistically significant.

**Results**

*Baseline characteristics*

In each hospital, 200 FEs without a working channel were collected. A total of 600 FEs were evenly distributed among the disinfection groups. Most of the FEs included in group 1 were Video Naso-Pharyngo-Laryngoscopes(63.7%), followed by Fiber Naso-Pharyngo-Laryngoscopes(33.0%) and pediatric fiber Naso-Pharyngo-Laryngoscopes(3.3%). The majority of FEs in group 2 consisted of Video Naso-Pharyngo-Laryngoscopes(62.3%), followed by Fiber Naso-Pharyngo-Laryngoscopes(35.0%) and pediatric Naso-Pharyngo-Laryngoscopes(2.7%).

*Results before disinfection*

After clinical use and manual pre-cleaning, a CFU count ranging from 0 CFUs to countless CFUs(i.e., >500 CFUs) was found(Table 1). Overall, 61/300(20.3%) FEs in group 1 and 38/300(12.7%) FEs in group 2 showed no contamination according to the culture method used after clinical use and manual pre-cleaning. After excluding the FEs that showed no contamination, a chi-square test was performed. There was no statistically significant difference in the distribution of FEs depending on the CFU count before disinfection between study groups(p = 0.19).

*Results after disinfection*

After disinfection with UV-C light, 195/239(81.6%) FEs showed 0 CFUs. After EWD disinfection, 187/262(71.4%) FEs showed 0 CFUs. Univariate logistic regression showed higher odds of obtaining 0 CFUs when using UV-C light disinfection(OR 1.78, 95% CI = 1.17-2.71; p<0.01). To identify relevant confounders for the relationship between the disinfection method and obtaining 0 CFUs, a multivariate logistics regression was performed, including participating hospitals and types of FE(Table 2). Again, UV-C light disinfection increased the odds of obtaining 0 CFUs compared to the EWD(OR 1.83, 95% CI 1.19-2.79; p<0.01), conditional on participating hospital and type FE.

Forty-four(18.4%) FEs were still contaminated after disinfection with UV-C light, with a CFU count ranging from 1 to 20 CFUs. Seventy-five(28.6%) FEs were still contaminated after disinfection with the EWD, with a CFU count ranging from 1 to 23 CFUs.

Five(2.1%) FEs in group 1 were more contaminated after disinfection(ranging from 1 to 5 CFUs) compared to prior disinfection(ranging from 0 to 4 CFUs). Twenty-three(8.8%) FEs in group 2 were more contaminated after disinfection(ranging from 1 to 23 CFUs) compared to prior disinfection(ranging from 0 to 15 CFUs).

**Discussion**

In this study, UV-C light was demonstrated to be more effective in reducing CFUs compared to the EWD when disinfecting FEs without a working channel. The study results were similar to thos of previousstudies (11, 19, 21, 22). In this study, 81.6% of the FEs without a working channel showed no contamination after UV-C light disinfection. Rudhart et al.(11) showed similar results: 86% of the FEs showed no contamination after disinfection with UV-C light. Our single-center study showed no contamination in 85.7% after UV-C light disinfection(19). Studies investigating UV-C light disinfection for other surfaces, including rigid endoscopes or hospital surfaces, showed no contamination in 90.0% and 80.0%(21, 22).

A CFU count was performed to express the reduction in microbiological contamination. In the Netherlands, the standard for bacteria exposed to the respective disinfection method under laboratory circumstances is a log 5 reduction and log 4 for fungi and viruses(23). However, the FEs were often contaminated with 1 to 50CFUs, making a log 4-5 reduction impossible. In literature, suggested cut-off points for the acceptable number of microorganisms on hospital surfaces are ≤2.5CFUs/cm^2^ or 5CFUs/cm^2^ on agar contact plates(24). Guidelines for surveillance and sampling FEs do not provide specific cut-off points for FEs without a working channel. The Duodenoscope Surveillance Sampling & Culturing protocol suggests establishing microbial cut-off limits for each facility (25). In this study, 0 CFUs after disinfection was considered safely disinfected.

To evaluate each disinfection methods equally without interference from chemicals used in manual pre-cleaning, the microbiological contamination was evaluated after manual pre-cleaning with only tap water. In vitro studies have already shown effective bacterial reduction after in vitro contamination and UV-C light disinfection (11, 21, 26, 27). However, this is not compatible with real-life situations, for example, because the surface on which the in vitro contamination was performed differs from the material of which the endoscope is made. We aimed to investigate whether UV-C light disinfection was equally effective as the EWD in a clinical setting. We tried to create a setting that was as realistic as possible.

A total of 5 FEs in the UV-C light group and 23 in the EWD group were more contaminated after disinfection than before disinfection. Potentially because the culture collection was performed incorrectly. Other explanations could be that the contamination occurred in the disinfector or during transportation to the sample collection. After disinfection using the EWD, the FEs were dried using a microfiber cloth. Directly afterward, a culture was collected. This was not a sterile process, so FEs were possibly contaminated during this process. However, it is most likely explained by the limited sensitivity of the chosen culture technique.

No gold standard exists for assessing microbiological contamination on the outer surface of FEs without a working channel. Several techniques have been described to determine surface contamination(28). Guidelines for reprocessing FEs usually also recommend a sampling method for determining the disinfection quality(7-10). However, most guidelines recommend sampling the fluid from the channels, which is not possible for FEs without a working channel. We performed a pilot study(submitted), showing that frequently described sampling techniques could all equally detect microbiological contamination after clinical use and pre-cleaning (data not shown, available upon request). The sampling techniques investigated were: rolling technique over a Trypticase Soy Agar with tween and lecithin plate, rolling technique over a Plate Count Agar+additives, swab technique, and broth technique. For practical reasons, the rolling technique over a Plate Count Agar+additives was chosen in this study. The rolling technique has also been used for sampling endoscopes without a working channel in previous studies and is considered a faster and more efficient method than dipping the tip in a solution(11, 21, 29).

In addition to the quality of the reprocessing process, efficiency, costs, and environmental impact are important. Disinfection with UV-C light significantly reduces the reprocessing time of FEs without a working channel, so fewer FEs and storage capacity are required. No water or chemicals are used for UV-C light disinfection except from the manual pre-cleaning, which reduces the environmental impact.

A limitation of this study is that only bacterial contamination was assessed. No statements can be made regarding eradicating non-bacterial pathogens, such as viruses, fungi, and prions. However, literature shows that UV-C light has the potential to inactivate viruses and fungi(30-32). The absorption spectra of peptide bonds in prions are possibly sensitive to UV-C light, but more research is needed(33). Secondly, a severe limitation of this study was that we did not perform a microbiological identification, making it impossible to draw conclusions regarding the bacteria found. A previous study analyzed bacterial contamination on rigid ORL endoscopes after UV-C light disinfection. Bacteria identified in this study could be attributed to the normal skin flora (coagulase-negative Staphylococcus and Micrococcus luteus)(21). In the study by Rudhart et al. microbiological contamination of FEs showed that bacteria found after UV-C light disinfection could be attributed to the normal mucosal microflora(Coagulase Negative Staphylococci, Micrococcus luteus, Bacillus spp. and Corynebacterium spp.)(11). Thirdly, in group 1, more FEs(20.3%) showed no contamination after clinical use and manual pre-cleaning compared to group 2(12.7%). Since the FEs were randomly assigned to one of the disinfection groups after clinical use, we do not have an explanation for the difference between the disinfection groups. Fourth, after using the EWD, FEs were dried with a microfiber cloth to remove excess water to prevent false bacterial contamination from the water used in the decontamination process. The water used for the EWD is routinely checked for contamination, so it is very unlikely that contamination due to contaminated water occurred. However, it cannot be ruled out completely. Since adequate hand sanitation occurred and the microfiber cloths were delivered aseptic, it is also unlikely that using a microfiber cloth resulted in contamination of the FE before being sampled for the second time. However, this cannot be completely ruled out.It is also possible that drying the FEs in a drying cabinet would have resulted in fewer contaminated samples after disinfection with the EWD. However, several guidelines recommend removing excess water with a clean, lint-free cloth after disinfection, for example, when the FE is to be used within 4 hours after disinfection(8). Taking samples after disinfection and removing excess water, as performed in this study, is still comparable to a real-life situation. Fifth, the culture collection was performed by two researchers(YH, MR), and the culture evaluation was done by either one of them or a laboratory analyst. This might have led to interobserver differences. Sixth, emerging evidence suggests bacteria enter a viable-but-nonculturable (VBNC) state after exposure to environmental stress, such as UV-C light disinfection (34, 35). We did not have the resources to investigate this in this study, so this should be further evaluated in future studies. Lastly, because UV-C light cannot penetrate dirt, debris, or grime, the effectiveness of UV-C light disinfection is highly dependent on the quality of manual pre-cleaning. Being a manual process, the quality of the pre-cleaning may vary from person to person. This could have led to a potential bias in our study. When implementing UV-C light disinfection, extra attention is required to ensure the quality of the pre-cleaning process.

The current study focused on disinfecting FEs without a working channel used in the ORL department. Future research should investigate whether UV-C light disinfection could also be applied to disinfect other medical instruments, such as endoscopes with a working channel (36).

**Conclusion**

In this study, UV-C light disinfection was more effective in reducing CFUs on FEs without a working channel than the standard disinfection method with the EWD. Therefore, disinfection with UV-C light appears to be a better method for bacterial disinfection for FEs without a working channel. However, the effectiveness of UV-C light disinfection strongly depends on the quality of the manual pre-cleaning process since UV-C light cannot penetrate debris.

**List of abbrivations**

FE = flexible endoscope

EWD = Endoscope Washer Disinfector

CFU = Colony Forming Unit

UV-C = Ultraviolet light group C

ORL = Otorhinolaryngology

ASGE = American Society for Gastrointestinal Endoscopy

ESGE-ESGENA = European Society for Gastrointestinal Endoscopy and European Society of Gastroenterology Nurses and Associates

HIPAC = Healthcare Infection Control Practices Advisory Committee

SFERD = Flexible Endoscopes Cleaning and Disinfection Steering Committee

MRSA = Methicillin-resistant Staphylococcus aureus

VRE = Vancomycin-resistant Enterococcus

**Declarations**

**Ethics approval and consent to participate**

The research ethics committee of all participating centers decided that this study would be carried out per the applicable legislation concerning reviewal by an accredited research ethics committee, such as Medical Research involving Human Subjects Act and Medical Treatment Contracts Act(file number 2021-9837)

**Consent for publication**

Not applicable

**Availability of data and materials**

The data used and analysed during the current study are available from the corresponding author on reasonable request.

**Competing interests**

Not applicable.

**Funding**

The author(s) disclosed receipt of the following financial support for the research, authorship and/or publication of this article: PENTAX Medical has funded the study. PENTAX Medical did not have a role in the study design, data collection, analysis or results.

**Authors' contributions**

YH and MR collected all the data. JK, RK and JZ performed the bacterial analysis. YH was the major contributor in writing the manuscript. MR, DW and HS contributed in writing the manuscript. All other authors read and approved the final manuscript.

**Acknowledgements**

Not applicable.

**References**

1. Kenters N, Huijskens EG, Meier C, Voss A. Infectious diseases linked to cross-contamination of flexible endoscopes. Endosc Int Open. 2015;3(4):E259-65.

2. Seoane-Vazquez E, Rodriguez-Monguio R. Endoscopy-related infection: relic of the past? Curr Opin Infect Dis. 2008;21(4):362-6.

3. Spaulding EH. Disinfection, sterilization and preservation. Chemical disinfect of medical and surgical materials1968. p. 517-31.

4. Veerabadran S, Parkinson IM. Cleaning, disinfection and sterilization of equipment. Anaesthesia & intensive care medicine. 2010;11(11):451-4.

5. Muscarella LF. High-level disinfection or "sterilization" of endoscopes? Infect Control Hosp Epidemiol. 1996;17(3):183-7.

6. Muscarella LF. Prevention of disease transmission during flexible laryngoscopy. Am J Infect Control. 2007;35(8):536-44.

7. Healthcare Infection Control Practices Advisory Committee. Essential Elements of a Reprocessing Program for Flexible Endoscopes–The Recommendations of the Healthcare Infection Control Practices Advisory Committee (HICPAC) 2016 [Available from: <https://www.cdc.gov/hicpac/pdf/flexible-endoscope-reprocessing.pdf>.

8. Day LW, Muthusamy VR, Collins J, Kushnir VM, Sawhney MS, Thosani NC, Wani S. Multisociety guideline on reprocessing flexible GI endoscopes and accessories. Gastrointest Endosc. 2021;93(1):11-33.e6.

9. Beilenhoff U, Biering H, Blum R, Brljak J, Cimbro M, Dumonceau JM, et al. Reprocessing of flexible endoscopes and endoscopic accessories used in gastrointestinal endoscopy: Position Statement of the European Society of Gastrointestinal Endoscopy (ESGE) and European Society of Gastroenterology Nurses and Associates (ESGENA) - Update 2018. Endoscopy. 2018;50(12):1205-34.

10. (SFERD) SFEReD. Kwaliteitshandboek flexibele endoscopen 2019.

11. Rudhart SA, Günther F, Dapper L, Thangavelu K, Geisthoff UW, Stankovic P, et al. UV light-based reprocessing of flexible endoscopes without working channel in Oto-Rhino-Laryngology: an effective method? Eur Arch Otorhinolaryngol. 2021;278(10):4075-80.

12. Hart D. Bactericidal ultraviolet radiation in the operating room. Twenty-nine-year study for control of infections. J Am Med Assoc. 1960;172:1019-28.

13. Rutala WA, Gergen MF, Weber DJ. Room decontamination with UV radiation. Infect Control Hosp Epidemiol. 2010;31(10):1025-9.

14. Marra AR, Schweizer ML, Edmond MB. No-Touch Disinfection Methods to Decrease Multidrug-Resistant Organism Infections: A Systematic Review and Meta-analysis. Infect Control Hosp Epidemiol. 2018;39(1):20-31.

15. Nerandzic MM, Cadnum JL, Pultz MJ, Donskey CJ. Evaluation of an automated ultraviolet radiation device for decontamination of Clostridium difficile and other healthcare-associated pathogens in hospital rooms. BMC Infect Dis. 2010;10:197.

16. Boyce JM, Havill NL, Moore BA. Terminal decontamination of patient rooms using an automated mobile UV light unit. Infect Control Hosp Epidemiol. 2011;32(8):737-42.

17. Jinadatha C, Villamaria FC, Restrepo MI, Ganachari-Mallappa N, Liao IC, Stock EM, et al. Is the pulsed xenon ultraviolet light no-touch disinfection system effective on methicillin-resistant Staphylococcus aureus in the absence of manual cleaning? Am J Infect Control. 2015;43(8):878-81.

18. Katara G, Hemvani N, Chitnis S, Chitnis V, Chitnis DS. Surface disinfection by exposure to germicidal UV light. Indian J Med Microbiol. 2008;26(3):241-2.

19. Halmans Y, Wellenstein DJ, Romijn M, Cremers S, Smit JJ, Hopman J, et al. Is ultraviolet light disinfection fit to be the future standard for the disinfection of flexible endoscopes without a working channel? Clin Otolaryngol. 2023.

20. Technologies US. Instructions for Use.

21. Rudhart SA, Günther F, Dapper L, Thangavelu K, Gehrt F, Stankovic P, et al. UV light-based decontamination: an effective and fast way for disinfection of endoscopes in otorhinolaryngology? Eur Arch Otorhinolaryngol. 2020;277(8):2363-9.

22. Casini B, Tuvo B, Cristina ML, Spagnolo AM, Totaro M, Baggiani A, Privitera GP. Evaluation of an Ultraviolet C (UVC) Light-Emitting Device for Disinfection of High Touch Surfaces in Hospital Critical Areas. Int J Environ Res Public Health. 2019;16(19).

23. Infectiepreventie SW. Reiniging, Desinfectie & Sterilisatie: Beleid [ZKH]. In: WIP-richtlijn, editor. 2011.

24. Cloutman-Green E, D'Arcy N, Spratt DA, Hartley JC, Klein N. How clean is clean--is a new microbiology standard required? Am J Infect Control. 2014;42(9):1002-3.

25. Duodenoscope Surveillance Sampling & Culturing. In: Collaboration DoHaHS, editor. 2018.

26. Kac G, Podglajen I, Si-Mohamed A, Rodi A, Grataloup C, Meyer G. Evaluation of ultraviolet C for disinfection of endocavitary ultrasound transducers persistently contaminated despite probe covers. Infect Control Hosp Epidemiol. 2010;31(2):165-70.

27. Chen GQ, Chen YH, Yi L, Yin J, Gao Q, Song JN, et al. Study on a New Ultraviolet Sterilizer to the Surface Disinfection of the Ultrasound Probe. Biomed Environ Sci. 2018;31(2):163-7.

28. Galvin S, Dolan A, Cahill O, Daniels S, Humphreys H. Microbial monitoring of the hospital environment: why and how? J Hosp Infect. 2012;82(3):143-51.

29. Maki DG, Weise CE, Sarafin HW. A semiquantitative culture method for identifying intravenous-catheter-related infection. N Engl J Med. 1977;296(23):1305-9.

30. Druce JD, Jardine D, Locarnini SA, Birch CJ. Susceptibility of HIV to inactivation by disinfectants and ultraviolet light. J Hosp Infect. 1995;30(3):167-80.

31. Yang JH, Wu UI, Tai HM, Sheng WH. Effectiveness of an ultraviolet-C disinfection system for reduction of healthcare-associated pathogens. J Microbiol Immunol Infect. 2019;52(3):487-93.

32. Vo E, Rengasamy S, Xu S, Horvatin M, Zhuang Z. New technique to evaluate decontamination methods for filtering facepiece respirators. Am J Infect Control. 2021;49(4):416-23.

33. Cobb TC. UV-C Decontamination: NASA, Prions, and Future Perspectives. Applied Biosafety. 2016;21(2):84-8.

34. Wilks SA, Koerfer VV, Prieto JA, Fader M, Keevil CW. Biofilm Development on Urinary Catheters Promotes the Appearance of Viable but Nonculturable Bacteria. mBio. 2021;12(2).

35. Highmore CJ, Warner JC, Rothwell SD, Wilks SA, Keevil CW. Viable-but-Nonculturable Listeria monocytogenes and Salmonella enterica Serovar Thompson Induced by Chlorine Stress Remain Infectious. mBio. 2018;9(2).

36. Mohsin MS, Avdic M, Fitzpatrick K, Lanzarini-Lopes M. UV-C side-emitting optical fiber-based disinfection: a promising approach for infection control in tight channels. Microbiol Spectr. 2024;12(6):e0004024.

**Tables**

Table 1: Number of flexible endoscopes (FEs) without a working channel depending on the number of Colony Forming Units (CFUs) before disinfection by disinfection group (p=0.02).

| Disinfection method | 0 CFUs | 1-50 CFUs | 51-500 CFUs | >500 CFUs | Total |
| --- | --- | --- | --- | --- | --- |
| UV-C light disinfection  *(% within disinfection method)* | 61 (20.3) | 163 (54.3) | 62 (20.7) | 14 (4.7) | 300 (100) |
| Endoscope Washer Disinfector (*% within disinfection method)* | 38 (16.5) | 191 (59.0) | 51 (18.8) | 20 (5.7) | 300 (100) |

Table 2: Multivariate logistic regression including all relevant covariates

| Parameter | OR | CI | P |
| --- | --- | --- | --- |
| Disinfection method UV-C light | 1.825 | 1.19-2.79 | <0.01 |
| Disinfection method Endoscope Washer Disinfector | Reference |  |  |
| Type of FE without a working channel | | | |
| Pediatric Naso-Pharyngo-Laryngoscopes | Reference |  |  |
| Video Naso-Pharyngo-Laryngoscopes | 1.02 | 0.18-5.73 | 0.98 |
| Fiber Naso-Pharyngo-Laryngoscopes | 1.15 | 0.34-3.86 | 0.83 |
| Participating hospital | | | |
| The Spaarne Hospital | Reference |  |  |
| Medical Spectrum Twente | 1.91 | 0.52-7.02 | 0.33 |
| Medical Center Leeuwarden | 1.16 | 0.26-4.73 | 0.83 |

**Appendix**

Appendix 1: Example of the microbiological culture collection using the rolling method.


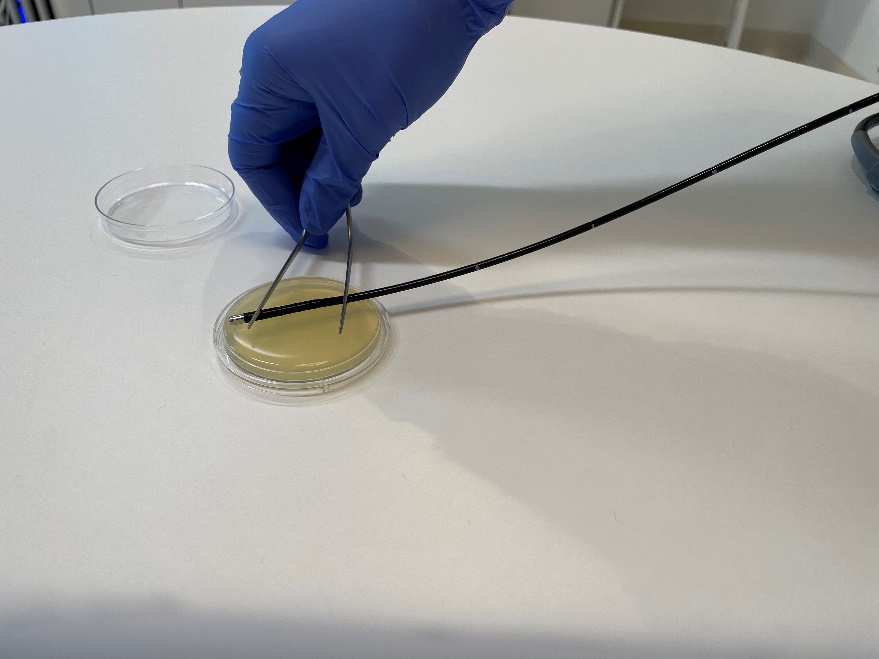


Appendix 2: Detailed protocol of the rolling method for culture collection of the flexible endoscope without a working channel

The rolling technique over a Plate Count Agar + additives:

1. Immediately after pharyngolaryngoscopy, transport the FE without a working channel to the designated room for manual pre-cleaning and sample collection in the following manner:
   1. The executor of the pharyngolaryngoscopy closes the transport container with the cover with the red smiley.
   2. The assistant collects the FE immediately and brings it to the assigned room for manual pre-cleaning and sample collection. The researcher will now start the procedure.
2. Manual pre-cleaning:
   1. Remove the cover with the red smiley face from the transport container.
   2. Perform hand disinfection and put on unsterile gloves.
   3. Moisten an unsterile 10x10cm gauze pad with tap water (not too wet! No water should drip when squeezing the moistened gauze).
   4. Wipe the moistened gauze over the FE using a rotary motion from proximal to distal. Repeat three times with a different portion of the gauze each time. Include the tip as well. If there is still visible debris left on the FE, repeat until all the debris is removed.
   5. Discard the gauze in the appropriate trash can and place the FE back in the transport container.
   6. Remove unsterile gloves and perform hand disinfection.
3. Collection the sample:
   1. Use the tip and the first 10cm of the distal part of the laryngoscope for the sample.
   2. Wear a medical face mask and unsterile gloves during the sample collection to avoid contamination. Make sure not to touch the tip and the first distal part of the FE except for sample collection.
   3. Perform hand disinfection and disinfect the surface the sample collection will take place on using disinfection wipes.
   4. Open the package of sterile tweezers, but do not remove the tweezers yet.
   5. Remove the lid from the Plate Count Agar + additives plate.
   6. Perform hand disinfection and put on unsterile gloves.
   7. Fix the distal part of the FE with the sterile tweezers to prevent partial lifting of the FE from the plate while making a rolling motion with the FE. Make sure that the entire circumference of the distal end of the FE touched the agar plate only once.
   8. Place the FE back in the transport container.
   9. Remove the unsterile gloves and perform hand disinfection.
   10. Put the lid back on the agar plate.
   11. Record serial number of the FE, study number, date and time of the sample collection and sample number and label the agar plate.
4. Place the labeled agar plate back in the heat incubator at 36 °C without the addition of CO₂ for 72 hours.
   1. Afterwards, perform a CFU count.
5. Transport the FE after the sample collection to the disinfection department:
   1. Close the transportation container with the cover with the red smiley face facing upwards.
   2. Transport the FE to the disinfection department and continue the disinfection process as protocolled by the hospital.
